# Exploring the behaviour of water in glycerol solutions by using delayed luminescence

**DOI:** 10.1371/journal.pone.0191861

**Published:** 2018-01-29

**Authors:** Rosaria Grasso, Francesco Musumeci, Marisa Gulino, Agata Scordino

**Affiliations:** 1 Department of Physics and Astronomy, Catania University, Catania, Italy; 2 Laboratori Nazionali del Sud, Istituto Nazionale di Fisica Nucleare, Catania, Italy; 3 Facoltà di Ingegneria e Architettura, Università di Enna Kore, Enna, Italy; Kermanshah University of Medical Sciences, ISLAMIC REPUBLIC OF IRAN

## Abstract

The crucial role of water in the engine of life have encouraged many researchers in studying, both theoretically and experimentally, the possible “structure” of water. Many properties of water have been related to the interplay between two distinct and interconverting structural species, namely the low-density water (LDW) and the high-density water (HDW). Supported by the results obtained with other aqueous solutions, this paper deals with the possibility of using the ultra-weak delayed luminescence (DL) to investigate water structuring in a mixture with glycerol, characterized only by hydrogen bonds between the various molecules. Spectral and temporal characteristics of DL decays give information on the two components of the mixture, by evidencing the contribution of water at glycerol concentrations close to the values used in cryopreservation. DL results have shown a correlation with LDW clusters size as determined by other researchers on the basis of neutron diffraction experiments and computational modelling, as reported in Literature.

## Introduction

The role of water and its anomalous properties in the chemistry of life have intrigued generations of scientists and the behaviour of water constitutes an open task up to now [1, 2]. Water is not just a solvent but it actively engages and interacts with biomolecules at nanoscale level in complex and essential ways for establishment and maintenance of life. The structure of hydration shells determines the biological functions of a protein and influences interaction with other protein or substrates [3, 4].

The extensive three-dimensional hydrogen bond network of H_2_O molecules plays a fundamental role in the behaviour of water. Even if the molecular movements in water require constant breaking and reorganization of individual hydrogen bonds on a picosecond time scale, at any instant, the degree of hydrogen bonding is very high showing a dynamic equilibrium among changing percentages of assemblages of different oligomers and polymer species (clusters), whose structure is dependent on temperature, pressure and composition.

It has been suggested that liquid water consists of two kind of micro-domains of rapidly exchanging polymorphism in dynamical equilibrium [5–12]: one form, namely the low-density water (LDW), with intermolecular hydrogen bonds like that of ordinary hexagonal ice, and the other one, namely the high-density water (HDW), with compact bonding similar to ice II. Several experimental results have supported the existence of two distinguishable structures in liquid water [13–17]. Moreover, a Debye-like slow relaxation was observed in water [18], associated to structural and/or dynamical inhomogeneity on length scale of the order of 100 μm, as the chainlike structures with a polymer-like dynamics proposed by Huang and co-workers [19]. In addition, a structural polymorphism of water (quasi-crystalline structures) has been suggested also in those salt solutions, where water structuring is expected, and in the solute-free zone (hundreds of μm in width and stable for days once formed) that water forms in proximity of various hydrophilic surfaces [13].

Due to the key role of water in biological systems, a better comprehension of water structuring is desirable. In this regard, the dynamics of complex systems such as hydrogen-bonding liquids and their mixtures is nowadays one of the most active areas of research. Particular interest there is in studying the action of glycerol on the structure of water. As it is known, as cryoprotectant glycerol acts by stabilizing macromolecules, cells and tissues under cooling to subzero temperatures, along with suppressing the formation of ice. The temperature behaviour of glycerol has thought to be due to the existence of an extended hydrogen-bond network. A recurring hypothesis in experimental and computational studies is that cryoprotectants act by modifying water structure [20–22].

Using a combination of neutron diffraction experiments and computational modelling in order to examine the hydrogen-bond networks in glycerol-water solutions at different concentration, it is reported in Literature that glycerol and water microsegregate to form hydrogen bonded clusters of water-rich and glycerol-rich regions, cluster sizes depending on the concentration [23–25]. Actually such segregation, that is the formation of nano-clusters of water surrounded by the matrix of solute molecules, was observed also with other cryoprotectants [26, 27].

It was found that at concentrations close to the value at which the mixture exhibits a minimum in the freezing point depression, the local density of water oxygen atoms in the water-rich region is significantly lower than the bulk value. It has been postulated that nanosegregation allows water to form a low-density structure that is protected by an encapsulating glycerol interface, so preventing ice formation [25].

In previous papers [28–30] the authors have studied the possibility of getting information on water structuring by performing delayed luminescence (DL) measurements from suitable water samples. More precisely, measurements were performed on super-cooled bi-distilled water at ambient pressure, in some aqueous salt solutions when on changing the temperature a displacement of the HDW/LDW equilibrium was foreseen [9, 31, 32], and in gel water-TEOS samples when, on aging, the gelation process led to the formation of a three-dimensional silica network.

It is called DL the ultraweak photo-emission occurring after the switching-off of the excitation source. The DL signal is prolonged in time going from few μs up to second or minutes. Firstly observed in plants [33], the general scheme for DL, also named delayed fluorescence, is known as the Jablonski diagram [34] and connects DL to the repopulation of the excited singlet state via back reaction from an undefined metastable state, where the energy is stabilized, so taking into account for long lasting times. Notwithstanding the low level of the signal, DL from biological samples has shown a strong connection to the state of the system. In particular, it has been demonstrated its connection with the integrity of the electron transport chain, as in Photosystem II of plant cells or in Complex I of mitochondrial respiratory chain of human cells. On the basis of obtained results application tools in plant breading [35–37], environment control [38], cancer research [39–43] and in-vivo measurements of mitochondrial oxygen tension [44] have been proposed. Moreover, studying the analogous behaviour of condensed matter and biological samples, it was assessed a correlation between the DL signals and the dynamic ordered structures of the samples [45]. In addition, to explain the characteristics of the DL signals a theoretical model was proposed, which connected the DL from biological samples with the excitation and decay of non-linear coherent localized electron states (excitons or solitons) in low dimensional biological macromolecules [46–48] which present chain-like structures. All together, these results suggest the possibility to use DL to investigate the structure ordering of water.

On the other hand, pure water also glows, but the intensity of luminescence is low, and it can be observed only with the use of highly sensitive photodetectors.

Different interpretation of the origin of the intrinsic water (and water ice) luminescence have been proposed [49–51], among which the hypothesis that the excitation energy is transmitted over a chain of hydrogen bonds to the luminescence emission centres, represented by defects in the structure of water [52]. Moreover, some kind of collective motion in hydrogen-bonded structures has been hypothesized [18] to explain the large time-scale relaxation observed in sub-mm liquid samples, and molecular dynamics simulations have shown that also optical phonon-like modes can propagate through the hydrogen-bond network over several nanometres even though the hydrogen-bonded chains are continuously broken [53].

Previously obtained results [28–30] have shown that DL signals from aqueous solutions were more significant when LDW clusters formation was prevalent, so in this paper we investigated the possibility to explore the behaviour of water in cryoprotectant glycerol-water solutions by measuring DL emission. In particular, a glycerol-water mixture in the concentration range close to the value corresponding to the lowest freezing temperature was considered. A comparison with higher and lower concentration values was performed. Emission spectra were evaluated by considering, due to the low level of the signal, three large but separate regions centred at the main wavelengths inside the whole range covered by the photomultiplier. The time resolved DL decays were modelled as compressed hyperbolas [54–55] and analytically de-convolved in order to obtain rate constant distributions. The present results not only confirm the previous ones obtained by the authors on other aqueous solutions, but they are in agreement with computer simulations performed by other authors too [25].

## Materials & methods

Ultra-pure water (Panreac Hiperpur-plus (TMA), MW 18,016), was used. Starting from pure Glycerol (Sigma-Aldrich G6279, MW: 92.09, Assay ≥ 99% (GC)), aqueous glycerol solution at different mole fraction x_g_ = 0.03, 0.09, 0.26 and 0.80 were prepared.

The Delayed Luminescence was measured by using the ARETUSA set-up, described in Ref [56]. A high-intensity pulsed nitrogen laser (Laserphotonic LN203C, λ = 337 nm, pulse width 600 ps, energy per pulse 100 ± 5 μJ) was used to illuminate the sample via a bifurcated optical fiber bundle (AVANTES FCR-7UV400-2-ME).

The solution sample was placed inside a cylindrical stainless steel holder (internal sizes: 50 mm in diameter, 90 mm in height, volume 177 ml) and filled the entire holder. The fiber tip was aligned with the cylinder axis, tilted of 45° with respect to the air-solution interface (in order to reduce internal reflection of light), and immersed at a depth of 3 mm. Starting from the measurement (in air) of the laser beam diameter at a fixed distance from the fiber aperture, half of the beam divergence angle θ_div_ was evaluated as 115 mrad. Such half divergence angle decreased, according to Snell law, to 85 mrad when water (refractive index 1.334) filled up the holder, and to 78 mrad when we used glycerol. The DL was collected by the same fiber, whose numerical aperture (NA = 0.22) allowed an acceptance angle θ_acc_ of the fiber of 222 mrad in the case of empty holder. The acceptance decreased to 164 mrad or 150 mrad when water or glycerol, respectively, filled up the holder. As a consequence the fiber collected photons from a solid angle that was about 3.7 times larger than the solid angle where the light was injected.

DL was detected in the wavelength range 400–750 nm by using a photomultiplier (PMT) working in single photon count regime and cooled down to -10°C. The photomultiplier tube (PMT) R7206-01 SEL was produced by Hamamatsu Photonics for photon counting applications and so it was able to work in digital mode. In ARETUSA system, the single pulse from PMT was discriminated, compared with a threshold level, shaped and then acquired by an EASY-MCS multichannel scaler (Ortec, AMETEK) as a function of the arrival time. The PMT was inhibited during the sample illumination by an electronic shutter that allowed measuring DL starting 10 μs after the switching off of the illuminating laser. The spectral analysis of DL signal was performed by using three Broadband Bandpass Interference Filters (Edmund Optics; centred at 450 nm, 550 nm and 650 nm; 50 nm FWHM). One filter at a time was placed between the PMT and the fiber by using a suitable wheel, housing the entire filters set.

DL measurements were performed at different temperatures starting from the highest temperature and cooling down the sample. To this aim the holder was kept in a thermal bath (Haake C25). Temperature of the thermal bath was lowered with a rate of about 0.5°C/min. The DL measurements were performed 20 min after the set temperature of coolant was reached. The temperature inside the sample was measured by using a diving Pt100 temperature sensor (TP472 I.0, accuracy ±0.25°C) connected with a thermometer (RTD HD2107.1, Delta OHM). The temperature inside the sample was uniform inside 0.1°C.

Data acquisition was performed by a devoted personal computer implemented with the ORTEC MCS plug-in card. The multi-channel scaler acquired the DL raw data in 50000 channels with a dwell time of 2 μs, so covering the time interval 10 μs—0.1 s.

At the end of this time interval the intensity of the emitted luminescence reached values comparable with the background data. To increase the level of the revealed signal, the counts of 500 repetitions (200 in the case pure glycerol) of the same run were added, with a laser repetition rate of 1 Hz.

In each run the emission from empty holder was evaluated and subtracted as background.

As it regards the evaluation of emission spectra the measured counts were compared after a renormalization procedure that took into account the spectral dependence of filters’ transmittance and PMT quantum efficiency.

To check the response of the apparatus an aqueous solution (0.1 mg/mL) of Rhodamine 123 (83702 Sigma-Aldrich) was used. The total emission in the time interval of one decay (0.1 s) in standard experimental conditions resulted 985 ± 14 counts as average and standard error of four independent experiments. DL emission spectrum of Rhodamine 123 aqueous solution at T = 20°C was evaluated and compared with standard Fluorescence emission spectrum as reported in Literature (see S1 Fig). Spectral total emission in the time interval of one decay (0.1 s) resulted 907 ± 5 counts in the wavelength range 525–575 nm and 65 ± 0.5 counts in the wavelength range 625–675 nm. Most emission was registered by using the filter centred at 550 nm, that is very close to the wavelength 430 nm where fluorescence maximum emission occurs. Taking into account the differences in DL and Fluorescence signal intensities, we can conclude that the two spectra are quite similar, which accords what it is reported in Literature about Delayed Fluorescence and Fluorescence emission spectra in plant at least [34].

To reduce random signal variations, inevitably linked to the low level of the signal especially at longer times, and reveal the underlying temporal trend of luminescence, a standard smoothing procedure [57] was used, by sampling the experimental points (channel values) in such a way that final data resulted equally spaced in a logarithmic time scale axis (see S2 Fig). DL intensity *I(t)* was expressed as the number of photons recorded within a certain time interval divided to the time interval and the number of repetitions. Final data are reported as average of no less than four independent measures of glycerol-water mixtures at the same concentration.

## Results

**Fig 1:**
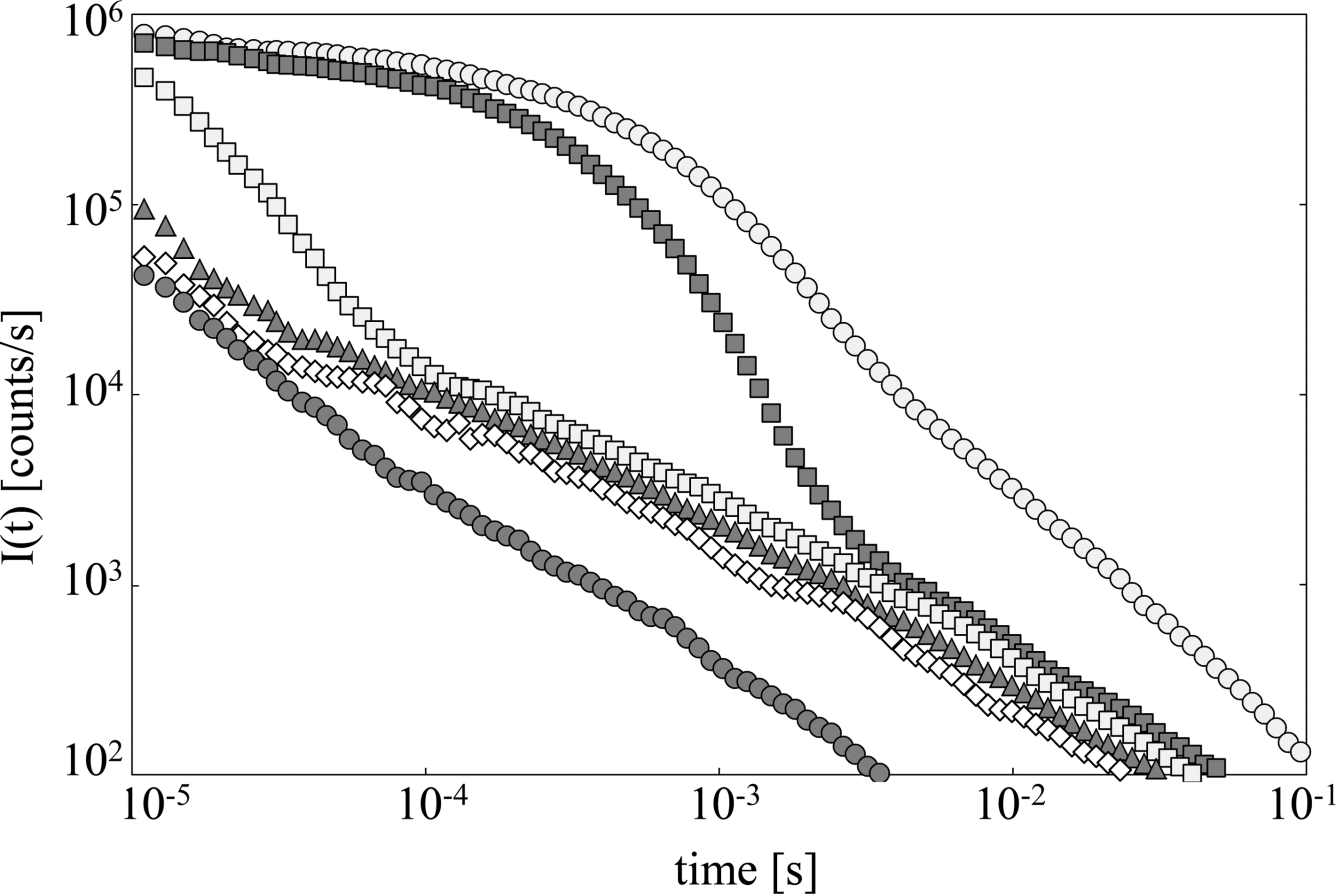
Time trends of delayed luminescence. Time trend of Delayed Luminescence, for different glycerol concentration (mole fraction, x_g_), at room temperature (20°C). (open circle) x_g_ = 1.00, (grey square) x_g_ = 0.80, (open square) x_g_ = 0.26, (grey triangle) x_g_ = 0.09, (open diamond) x_g_ = 0.03, (grey circle) x_g_ = 0.00, pure water. Data are average values. Standard errors are within the markers size.

Fig 1 reports time trends of DL intensity *I(t)*, detected by the photomultiplier when no spectral filter was used, emitted from samples at different glycerol concentration (mole fraction, x_g_). Relevant changes, both in the intensities and in the dynamics, appear moving from the glycerol sample (x_g_ = 1.00) to pure water (x_g_ = 0.00).

**Fig 2:**
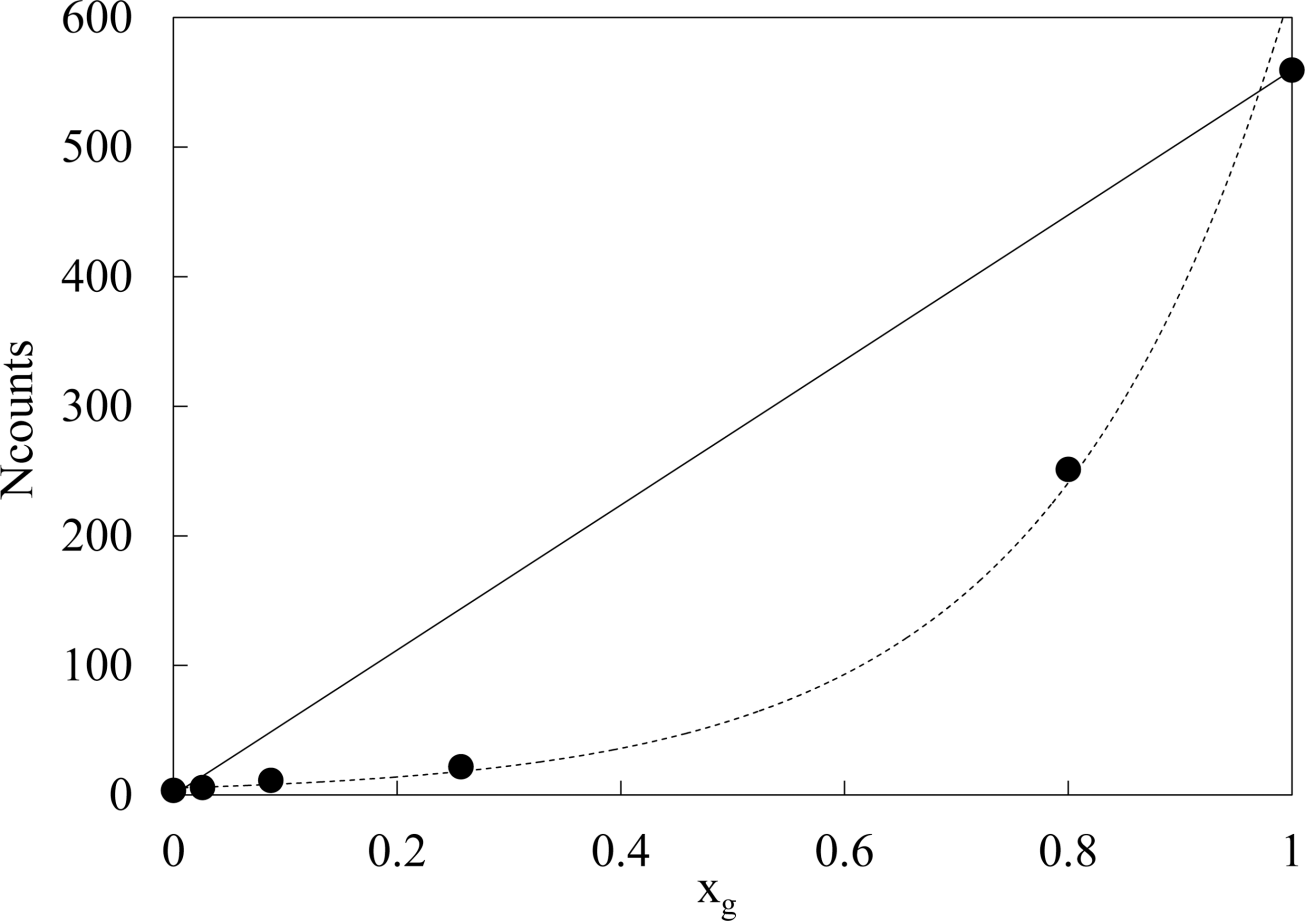
Total number of counts vs glycerol mole fraction. Total number of counts *N*_*counts*_ (●) registered in the acquisition time interval as a function of glycerol mole fraction x_g_. Solid line refers to a linear trend. Dashed line refers to the exponential trend Eq 2 that accords experimental data with R^2^ = 0.985. Standard errors are within the markers size.

Starting from the experimental intensity data *I*_*exp*_*(t)* it has been possible to evaluate the total numbers *N*_*counts*_ of DL counts in the time interval of the decay:

$$N_{counts}=\int_{t_{i}}^{t_{f}}I_{exp}(t)dt$$ (1)

where *t*_*i*_ and *t*_*f*_ are the initial and final time instants of the experimental decay, respectively. *N*_*counts*_ is connected to the total number of states excited by illumination that radiatively decay. Fig 2 reports *N*_*counts*_ as a function of the glycerol mole fraction. Experimental points move away from a linear trend noticeably. A suitable exponential trend

$$N_{counts}=a\;e^{b\;x_{g}}$$ (2)

accords experimental points (R^2^ = 0.98) with *a* = 5.4±0.8 and *b* = 4.7±0.3.

**Fig 3:**
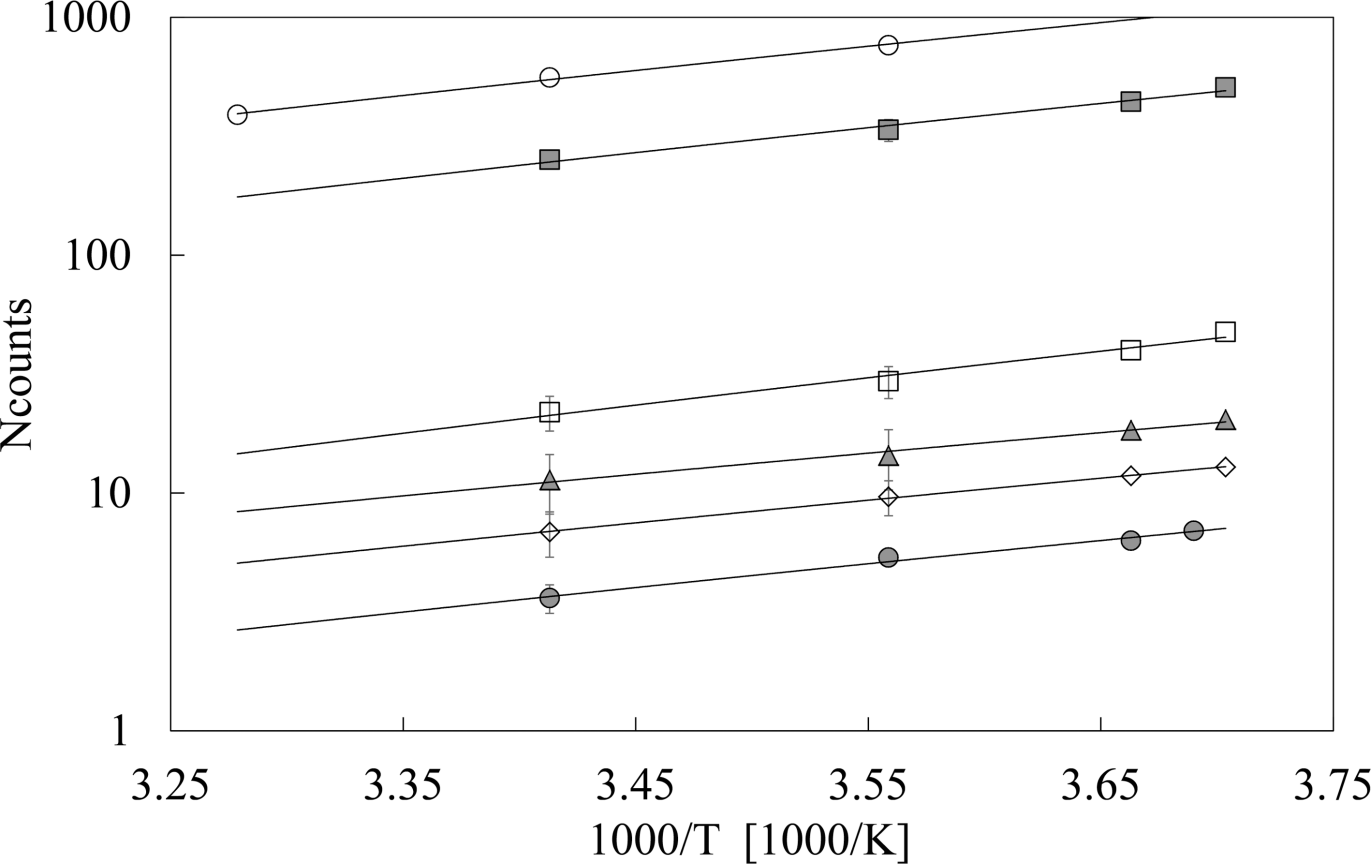
Total number of counts vs temperature. Arrhenius plot of the total number of counts *N*_*counts*_ registered in the acquisition time interval for different glycerol mole fraction x_g_: (open circle) x_g_ = 1.00, (grey square) x_g_ = 0.80, (open square) x_g_ = 0.26, (grey triangle) x_g_ = 0.09, (open diamond) x_g_ = 0.03, (grey circle) x_g_ = 0.00, pure water. If not explicitly reported, errors are within the markers size.

In Fig 3 the values of *N*_*counts*_ are reported for each mole fraction x_g,_ as a function of inverse of the temperature in the range -3 ÷ 20°C for pure water and glycerol-water solutions, and in the range 8 ÷ 32°C for pure glycerol. It appears that on decreasing the temperature DL signal increases whatever glycerol molar fraction. Data are well accorded (R^2^ ≥ 0.98) by Arrhenius trends, that are shown in figure as solid lines, whose slopes are quite independent, within experimental errors, on the glycerol mole fraction. The corresponding activation energies, evaluated from the trends gradient, gave as result an average value *ΔE* = 20±2 kJ mol^-1^.

**Fig 4:**
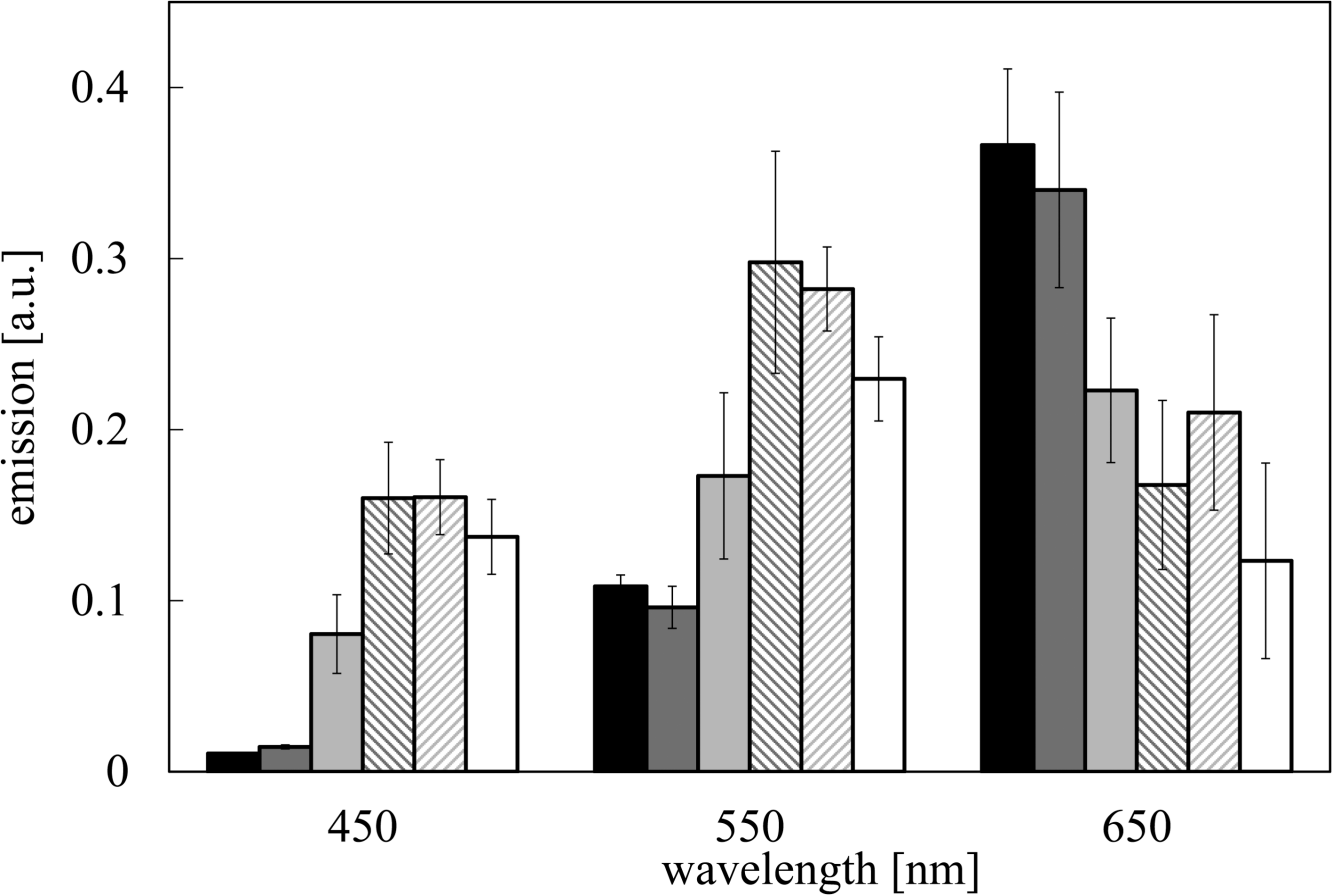
Emission spectra of delayed luminescence. DL emission spectra from samples of different mole fraction x_g_: (black) x_g_ = 1.00, (dark grey) x_g_ = 0.80, (grey) x_g_ = 0.26, (backward slash) x_g_ = 0.09, (slash) x_g_ = 0.03, (white) x_g_ = 0.00 (water). T = 20°C. Experimental data are normalized taking into account the spectral dependence of filters’ transmittance and PMT quantum efficiency. Average values and standard deviations are reported.

Emission spectra of the sample were registered in three spectral ranges centred at λ_em_ = 450 nm (blue component), λ_em_ = 550 nm (yellow component) and λ_em_ = 650 nm (red component), respectively (see Material & Method). Spectra are reported in Fig 4. Each component was normalized to the total emission registered by PMT, taking into account the PMT quantum efficiency. Data reported in Fig 4 refers to the room temperature condition. No change in the spectra was observed on varying the temperature in the interval -3 ÷ 20°C (see S3 Fig). Relevant differences occur by comparing pure glycerol (x_g_ = 1.00) and water (x_g_ = 0.00) spectra. In the case of glycerol the red component is the largest one, while in the case of water the maximum of emission occurs at shorter wavelengths, in the spectral region around 550 nm. At higher glycerol mole fraction (x_g_ > 0.26) spectra coincide with the glycerol one, while at lower values (x_g_ < 0.26) spectra coincide with the water one, inside the experimental errors. Interestingly, at glycerol mole fraction x_g_ = 0.26, that is very close to the concentration value at which the mixture exhibits a minimum in the freezing point depression, the spectrum is markedly different from the glycerol one, with maximum moving towards shorter wavelength.

The time trends as that reported in Fig 1 show a multimodal behaviour that can be modelled by the sum of a few Becquerel functions (compressed hyperbolas), appropriately weighted, as used to describe decays of complex systems [54, 55]. Worth to note that such power law functions could represent the convolution of a suitable continuum distribution of exponential decays, in turn [58]. The examined data were well fitted by a bimodal function according to the equation:

$$I(t)=\frac{I_{0_{fast}}}{{\left(1+t/t_{0_{fast}}\right)}^{m_{fast}}}+\frac{I_{0_{slow}}}{{\left(1+t/t_{0_{slow}}\right)}^{m_{slow}}}$$ (3)

where the *I*_*0i*_, *t*_*0i*_ and *m*_*i*_ parameters can be determined by non-linear least square methods. In this case the Excel Solver function was used, by imposing suitable constrains to the parameters.

**Fig 5:**
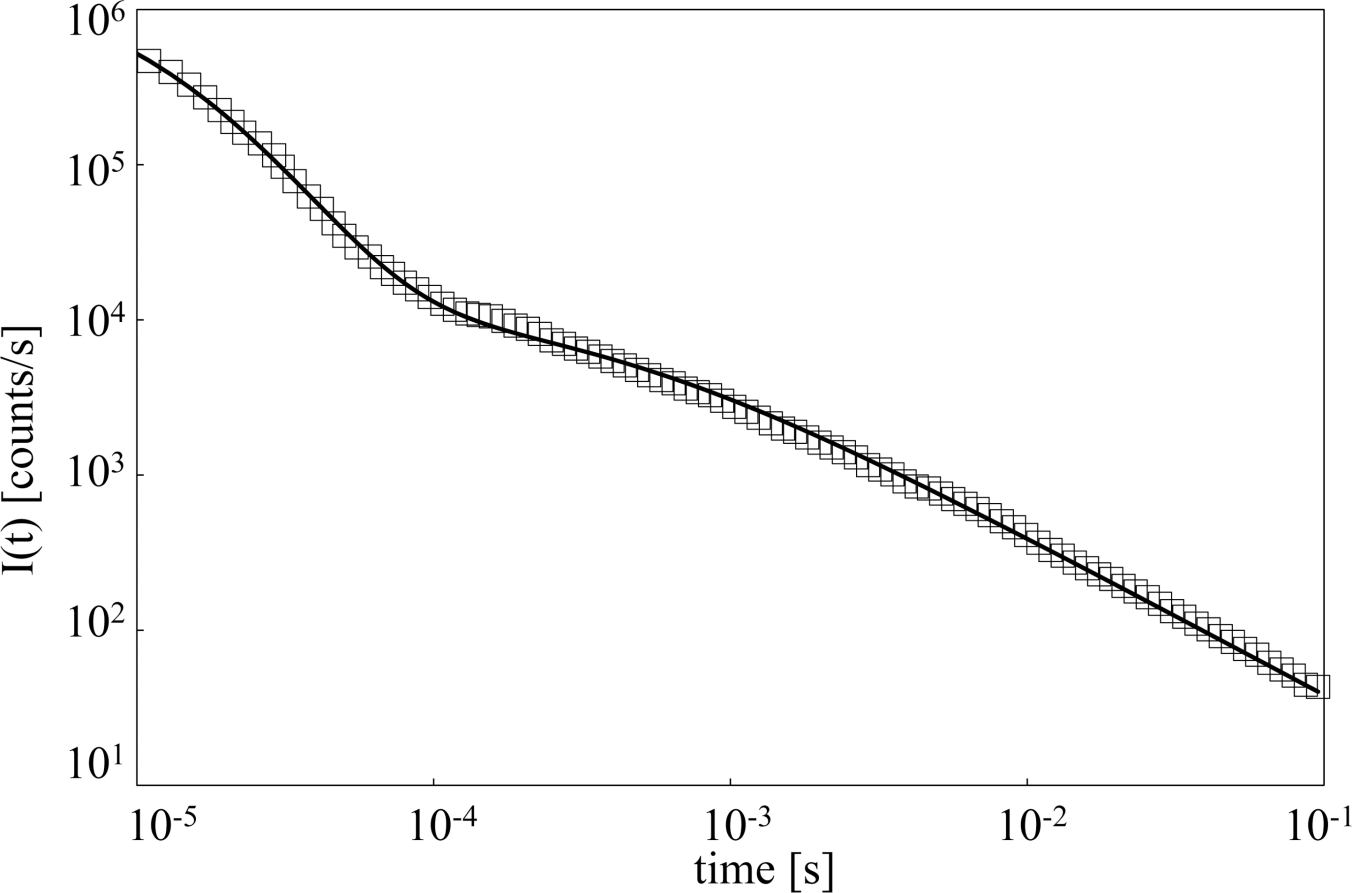
Theoretical fit of delayed luminescence time trend. DL time trend of a sample at glycerol mole fraction x_g_ = 0.26: (marker) experimental points; (solid line) fitting curve according to Eq 3.

Fig 5 shows the decay *I*_*exp*_*(t)* (marker) for the sample at mole concentration x_g_ = 0.26, along with the theoretical trend *I(t)* according to Eq 3. The average ratio between residual and datum is 0.05.

For each sample, the best fit (reduced χ^2^ ≤ 1) was obtained by considering one component with high decay rate, namely a *fast-component* with *m*_*fast*_ > 2, and one component with low decay rate, namely a *slow-component* with *m*_*slow*_ ≈ 1. Accordingly it was settled *t*_*0fast*_ < *t*_*0slow*_, their time range depending on the sample. More precisely *t*_*0fast*_ and *t*_*0slow*_ were in the millisecond range in the case of samples at x_g_ = 1.00 and x_g_ = 0.80, while for all the other samples (x_g_≤ 0.26), including pure water, it resulted *t*_*0fast*_ ≈ 0.01 ms and *t*_*0slow*_ ≈ 0.1 ms. According to the data shown in Fig 1, the *fast-component* is predominant in pure glycerol, while the *slow-component* is predominant in pure water.

As above said, the time relaxation of complex systems is usually described in terms of convolution of a continuous distribution of decay rate constants as follows

$$I(t)=A\int_{0}^{\infty }p(\gamma )e^{-\gamma t}d\gamma$$ (4)

where the distribution function *p(γ)* expresses the probability of an exponential decay with lifetime τ = 1/*γ*, and $\int_{0}^{\infty }p(\gamma )d\gamma =1$.

As a result, *I(t)* is the Laplace transform of the distribution function *p(γ)*. In order to determine the distribution function *p(γ)*, the inverse Laplace transform of *I(t)* must be performed. If data are accorded by a power law as those used in Eq 3 such an inversion can be analytically performed, because in this case the inverse Laplace transform is represented by a Gamma function [58]. Accordingly, in our case the sum of two Gamma distributions represented the rates distribution function *p(γ)*. Due to the large variability of *γ* values, it was suitable to study the function *p’(γ)* which represents the rate distribution function defined in a logarithm *γ* scale, such that *p(γ)*d*γ = p’(γ)*d*log*(*γ)*, characterized by a maximum at the rate constant γ_max_ [55]:

$$\gamma_{max}=\frac{1}{\left(I_{0_{fast}}+I_{0_{slow}}\right)}\left(I_{0_{fast}}\frac{m_{fast}}{t_{0_{fast}}}+I_{0_{slow}}\frac{m_{slow}}{t_{0_{slow}}}\right)$$ (5)

**Fig 6:**
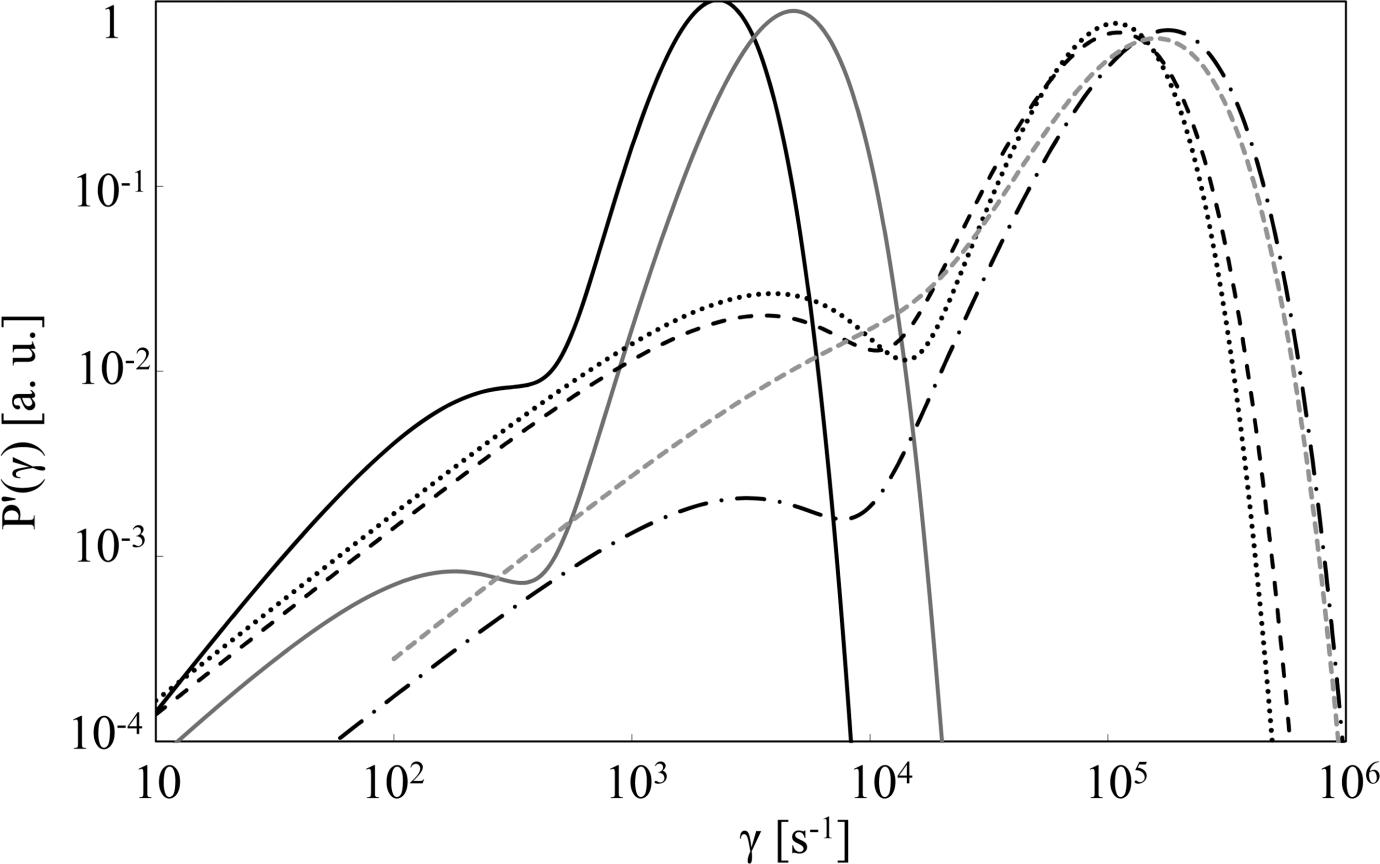
Rate distribution functions for DL decays. Rate distribution functions for DL decays from samples at different glycerol mole fraction x_g_: (black solid line) x_g_ = 1.00, (grey solid line) x_g_ = 0.80, (dash-dotted line) x_g_ = 0.26, (dashed line) x_g_ = 0.09, (dotted line) x_g_ = 0.03, (grey dashed line) x_g_ = 0.00 (water). T = 20°C.

Fig 6 shows the distribution function *p’(γ)* for the different mixtures. The marked difference between samples at high and low glycerol mole fraction x_g_ turns out in a distinct range of γ values. So we assumed the presence in DL decay of two components, one mostly related to glycerol and the other to water.

Evaluating the contribution of each component to the DL total emission as:

$$N_{fast}=\int_{t_{i}}^{t_{f}}\frac{I_{0_{fast}}}{{\left(1+t/t_{0_{fast}}\right)}^{m_{fast}}}dt;\;N_{slow}=\int_{t_{i}}^{t_{f}}\frac{I_{0_{slow}}}{{\left(1+t/t_{0_{slow}}\right)}^{m_{slow}}}dt$$ (6)

and taking into account the different mole fractions of glycerol and water in the samples, we defined a DL yield of each contribution as:

$${DL}_{fast}=\frac{N_{fast}}{glycerol\;mole\;fraction};\;{DL}_{slow}=\frac{N_{slow}}{water\;mole\;fraction}$$ (7)

**Fig 7:**
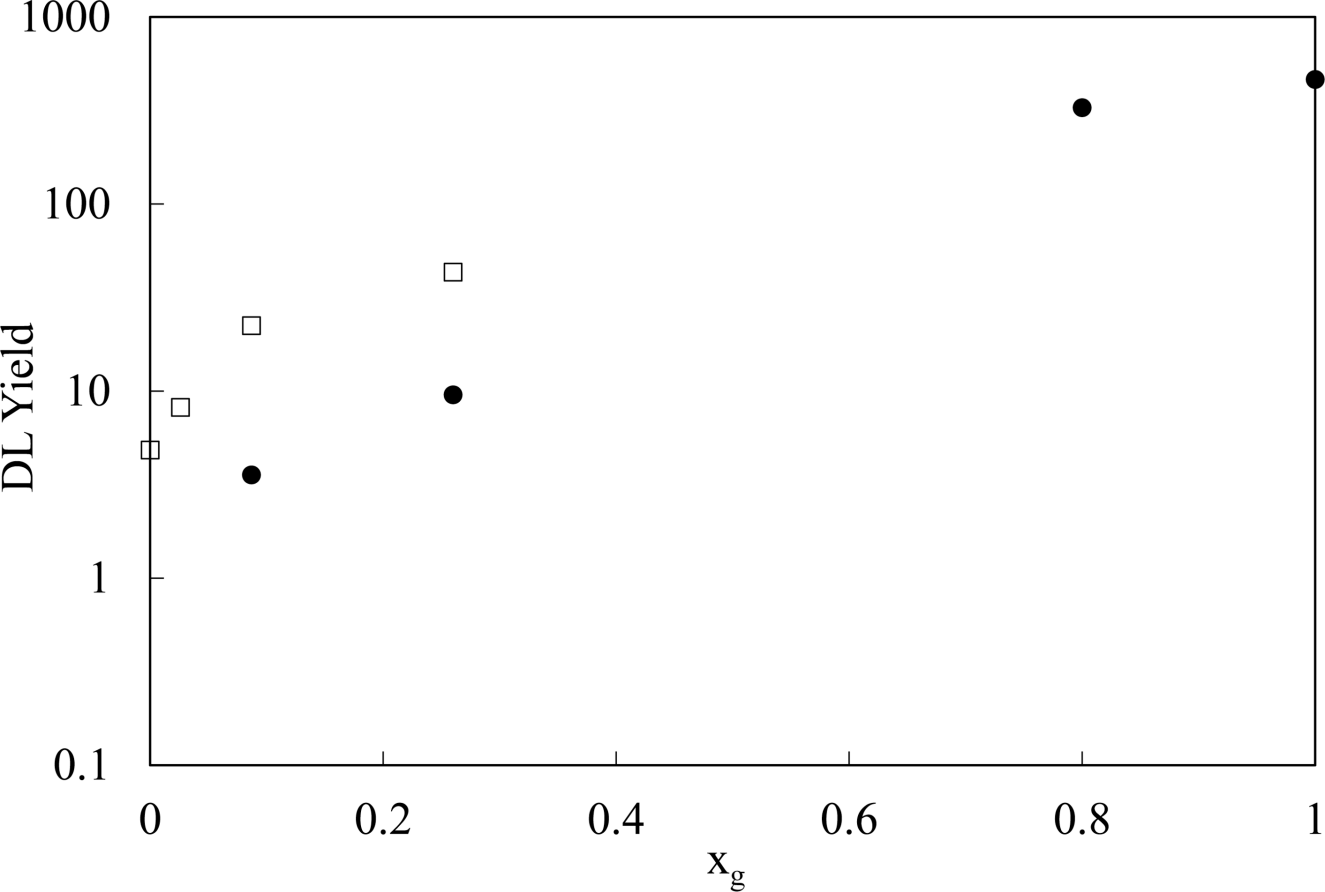
Delayed luminescence yield vs glycerol mole fraction. DL yield, according Eq 7, as a function of glycerol mole fraction x_g_: (●)022f5cg *DL*_*fast*_, (□) *DL*_*slow*_. Standard errors are within the markers size.

Plot of *DL*_*fast*_ and *DL*_*slow*_ is reported in Fig 7 as a function of glycerol mole fraction x_g_. No significant value (within the errors) was detected for *DL*_*fast*_ of samples at x_g_ = 0.03 and *DL*_*slow*_ of samples at x_g_ = 0.80. It appears that, while *DL*_*fast*_ data maintain an exponential trend (R^2^ ≥ 0.98) as that of Eq 2, a linear relationship (R^2^ ≥ 0.98) with x_g_ can be drawn for the *DL*_*slow*_ data set.

## Discussion

Luminescence of Glycerol after UVA laser excitation is very high with respect the water one, as reported in Figs 1–3. Moreover DL emission spectra of glycerol and water are quite different, being the red component (λ_em_ = 650 nm) the highest one in the case of glycerol, while the maximum moves towards shorter wavelengths in the case of water and glycerol–water mixtures. This result, along with the nonlinear dependence of *N*_*counts*_ as a function of glycerol mole fraction (see Fig 2) allows to assess that change in DL signal from aqueous glycerol solutions cannot be simply interpreted as a dilution effect, that should be represented by a linear decrease of *N*_*counts*_ on decreasing the glycerol content as sketched in Fig 2. In Literature nonlinear trend with glycerol concentration was observed in the viscosity of aqueous glycerol solutions [59–60]. Viscosity data showed an increase on increasing the glycerol concentration with a trend much steeper than the one reported in Fig 2 for DL data. As a matter of fact, by according data from Ref [59] to Eq 2 it results *b* = 8.1±0.5 (R^2^>0.98), and by using the larger set of data from Ref [60] one get *b* = 7.2±0.2 (R^2^>0.98). Both values are about twice the *b* value found by according *N*_*counts*_ data to Eq 2. In order to evaluate possible impact of the diffusion of excited species away from the optical window of the detector fiber, the diffusion length *d* = $\sqrt{2Dt}$ was calculated by using data of diffusion coefficients *D* reported in Literature [61] and considering the duration of a DL measurement (t = 0.1 s). As an exemplum, by considering the water-glycerol mixture at glycerol mole fraction x_g_ = 0.077, we get a diffusion coefficient *D* ≅ 0.6×10^−9^ m^2^/s [61], that gives rise to a diffusion length d ≅ 11μm. Alternatively, starting from the viscosity data reported in Ref [59], the diffusion coefficient *D* can be evaluated by using the Stokes-Einstein relation. Assuming 0.2 nm particle radius (water molecule) we get, at x_g_ = 0.115, *D*≅ 0.3×10^−9^ m^2^/s and consequently *d* ≅ 8μm. The evaluated diffusion lengths are much smaller than the fiber size (400 μm). So, taking also into account that, as specified in M&M, the acceptance solid angle (detection path) is 3.7 greater than the illuminated (excitation path) solid angle, we can exclude that the non-linear decrease of *N*_*counts*_ with concentration could be related to viscosity and consequent diffusion processes. Such decrease actually reflects an interplay between glycerol and water, where the structure of each of them play an important role in determining luminescence emission. So luminescence from water, that is related to the structure of water, must be taken into account. Indeed Lobyshev et al. [52] observed an intrinsic luminescence from water, whose emission spectrum had a peak in the blue region (more precisely at 410 nm) if excited at 310 nm. After investigating also the luminescence from high diluted aqueous solutions of “luminescent” and “non luminescent” substances, they assessed that “although the lifetime of a single hydrogen bond is very short, water at each moment of time constitutes an infinite structure containing ordered clusters and disordered regions like a crystal with defects”. In such a way, the hypothesized dynamic quasi-lattice of water should support the transfer, over a chain of hydrogen bonds, of the absorbed energy from the excitation site to the defects which act as source of luminescence.

Actually, the temperature dependence of *N*_*counts*_ corresponds to an activation energy *ΔE* = 20±2 kJ mol^-1^, that is smaller but very close (within the errors) to the value *ΔE* = 23±2 kJ/mol^-1^ obtained in the case of aqueous solutions of silica gel [30], where DL changes were attributed to water structuring triggered by the oxidic network. Such energy values, as the lower ones obtained in the case of some salt solutions in bi-distilled water (about 14±2 kJ mol^-1^) [28], are within the energy range that characterizes the bending or breaking of hydrogen bond [1]. So we can assume that *ΔE* should represent the energy required to promote the displacement of the LDW/HDW equilibrium [6, 7].

The analysis of the decay rate distributions in Fig 6 shows that the maximum value, for water and glycerol-water solutions (at x_g_≤ 0.26) occurs at γ_max_ (Eq 5) in the range of 10^5^ s^-1^, that is in good accord with the value of the lifetime τ_0~_5 μs corresponding to the maximum amplitude in the lifetime spectrum of the DL decays from both salt [28] and silica gel [30] aqueous solutions. Actually the values of *t*_*0fast*_ and *t*_*0slow*_ Eq 3 found for the all samples at x_g_≤ 0.26 are about the same values used to accord the DL time decays of silica gel solutions to a trend as Eq 3 [30].

Taking into account the results reported in Figs 1, 2, 4 and 6, we can assess that it is possible to distinguish two different contributes due to, namely, glycerol and water that do not combine, according to Fig 2, proportionally to their mole fractions. In Literature [23–27] it is reported that the presence of glycerol affects water structuring. Our results confirm that photon emission is affected too. The impact of glycerol on water structure has been largely examined in Literature [23–27] due to the importance of its use as cryoprotectant in the storage of biological molecules. In particular studies performing neutron scattering analysis combined with computer modelling [23–27] have allowed to propose the confinement of water in the matrix of the cryoprotectant as the main reason for the lack of ice formation. It was found that glycerol and water form hydrogen bonded glycerol-rich and water-rich clusters, postulating that such nanosegregation allows water to form a low density structure that is protected by an extensive and encapsulating glycerol interface. Moreover in Literature [25] it is reported that the glycerol-rich and water-rich clusters are unchanged upon cooling. According to Towey and co-workers [25] the temperature instead affects the tetrahedrality of the water network that can be quantified by measuring the included angle formed by three water oxygen atoms. They found an increase in the proportion of water oxygen triplets that have an angle near a perfect tetrahedral angle (109.5°) for the mixtures at x_g_ = 0.1 and x_g_ = 0.25 on decreasing the temperature.

The fact that the extension of glycerol and water regions does not depend on the temperature could explain why we do not observe significant changes in the DL emission spectra on varying the temperature with respect the spectrum reported in Fig 4. Instead the increased tetrahedrality on decreasing the temperature could give rise to a more ordered water structure that could take into account for the increase in the DL total emission *N*_*counts*_ on cooling, as reported in Fig 3, confirming once again the correlation between the DL and the order of the emitting structure, as showed in a previous research [45].

Interestingly, by studying the DL component related to water, i.e. the “slow” component, and normalizing *N*_*slow*_ total emission to the water mole fraction in order to get a parameter which does not depend on the quantity of water, we found that the DL yield per mole, *DL*_*slow*_, has a maximum at x_g_ = 0.26. The behaviour of *DL*_*slow*_ as a function of glycerol mole fraction x_g_, as reported in Fig 7, can be correlated to the cluster size distributions for water molecules as simulated by Towey and co-workers [25]. As a matter of fact, by defining water cluster size as the number of water molecules that participate in a continuous hydrogen-bonded network, Ref [25] reported that the average water cluster size was greater in the glycerol-water mixture at x_g_ = 0.25 than in the one at x_g_ = 0.1, while the most water molecules existed as isolated molecules in the glycerol-water mixture at x_g_ = 0.80. This correlates with the value of *DL*_*slow*_ that is greater for the sample at x_g_ = 0.26 than for the one at x_g_ = 0.09 (see Fig 7), and it is non-detectable at x_g_ = 0.80.

Further comparison can be drawn between our data and simulation results of Ref [25] as described in the following. Data reported in Fig 7 shows that *DL*_*slow*_ yield for the sample at x_g_ = 0.26 is 1.9 time greater than *DL*_*slow*_ yield for the one at x_g_ = 0.09. A previous research [45] showed a dependence of DL emission, from both biological and condensed matter materials, on the linear dimensions of the ordered emitting structure. So, the obtained *DL*_*slow*_ yield values suggest that the ratio between the average linear dimensions of water clusters in the two glycerol-water mixtures should be 1.9. For comparison, by considering the cluster size distribution reported Fig 3B of the paper by Towey and co-workers [25] the average cluster size for each mixture can be roughly evaluated. Taking into account that such size denotes the number of involved water molecules, its cube root value is related to the average linear space extension of the corresponding LDW clusters. It results that the ratio of such linear extension values between x_g_ = 0.25 and x_g_ = 0.1 glycerol-water mixtures is 1.7. This value well accords the above evaluated ratio value for *DL*_*slow*_ yield especially if one takes into account that the range interval of x_g_ used for *DL*_*slow*_ yield is slight larger than the one used in the computer simulation reported in Ref [25].

In addition, even if data reported in Ref [25] showed that the water networks in both the mixtures at x_g_ = 0.1 and x_g_ = 0.25 had water oxygen triplets with an angle actually much closer to the perfect tetrahedral one at low temperature than at room temperature (the large increase was seen for the mixture at x_g_ = 0.25), it must be noted that also at room temperature such mixtures showed a tetrahedrality greater than the liquid water cooled to 280K, being the mixture at x_g_ = 0.25 the one with the water oxygen triplets angle closest to the perfect tetrahedral angle. The increased tetrahedrality of the mixtures could also contribute to the increase in *DL*_*slow*_ yield with x_g_ shown in Fig 7, as above explained.

Overall these results accord the previous results obtained with salt and silica gel aqueous solutions [28–30], extending them to a system characterized only by hydrogen bonds between the various molecules.

## Conclusions

In conclusion, we have investigated water and water-glycerol mixtures, which are used in the cryopreservation processes, checking the influence of glycerol on the intrinsic delayed luminescence of water at different temperatures.

The spectral and temporal analysis of DL emission characterizes differently the behaviour of glycerol and water, allowing to distinguish the contribution of the two components to the mixture. Our measurements confirm the existence of intrinsic DL from water with a typical lifetime distribution ranging from microsecond up to hundreds of millisecond.

In this paper, we were able to correlate the DL emission from water in the mixture to the size of LDW clusters, as other authors evaluated by computer simulation. In particular, we found that the maximum of DL yield, as well as the maximum size of such clusters, occurs in correspondence to the concentration value at which the mixture water–glycerol exhibits a minimum in the freezing point.

On the other hand the many physical and chemical properties of water, that have a crucial and unique role for life as we know it, are related to such low density structuring. In this respect, the possibility to explore the water structuring by using DL measurements could constitute a powerful tool of investigation of life’s processes.

Nevertheless, these results are in some way puzzling: how it is possible that the excited states generating the DL exhibit so long lifetimes? Such a states come out from the hydrogen bond network existing between water and glycerol molecules and their lifetimes are more than six order of magnitude larger than the supposed lifetime of single hydrogen bond. A possible solution is to hypothesize the presence of collective states able to survive as topological singularities for very long times. We are planning further measurements to answer this question because it could open a new landscape in the understanding of both the behaviour and role of water in the functional organization of living systems.

## Supporting information

S1 Fig Emission spectra of Rhodamine 123. (Dashed line) Delayed Luminescence emission spectrum of an aqueous solution of Rhodamine 123 (0.1 mg/mL). Experimental data are normalized taking into account the spectral dependence of filters’ transmittance and PMT quantum efficiency. Average of four independent experiments ± SE. The total emission in the time interval of one decay (0.1 s) was evaluated as 985 ± 14 counts. For comparison (solid line) the Fluorescence emission spectrum of Rhodamine 123 dissolved in ethanol, when excited at 480nm, it is reported [elaborated from: http://omlc.org/], normalizing every point to the maximum intensity value at λ_em_ = 530 nm. Taking into account the differences in DL and F signal intensities, the spectra are quite similar.
(TIF)

S2 Fig Effect of smoothing procedure. Statistical variations of DL decay from glycerol sample (x_g_ = 1) at 20°C and underlying temporal trend after smoothing procedure.
(TIF)

S3 Fig Emission spectra of delayed luminescence at -3°C and 8°C. DL emission spectra from samples of different mole fraction x_g_: (black) x_g_ = 1.00, (dark grey) x_g_ = 0.80, (grey) x_g_ = 0.26, (backward slash) x_g_ = 0.09, (slash) x_g_ = 0.03, (white) x_g_ = 0.00 (water). (a) T = -3°C, (b) T = +8°C. Experimental data are normalized taking into account the spectral dependence of filters’ transmittance and PMT quantum efficiency. Average values and standard deviations are reported.
(TIF)
