# Supplementary figures and images for: Exploring the behaviour of water in glycerol solutions by using delayed luminescence
Source: PLoS One. 2018 Jan 29;13(1):e0191861. doi: 10.1371/journal.pone.0191861 (PMC5788358; doi:10.1371/journal.pone.0191861)

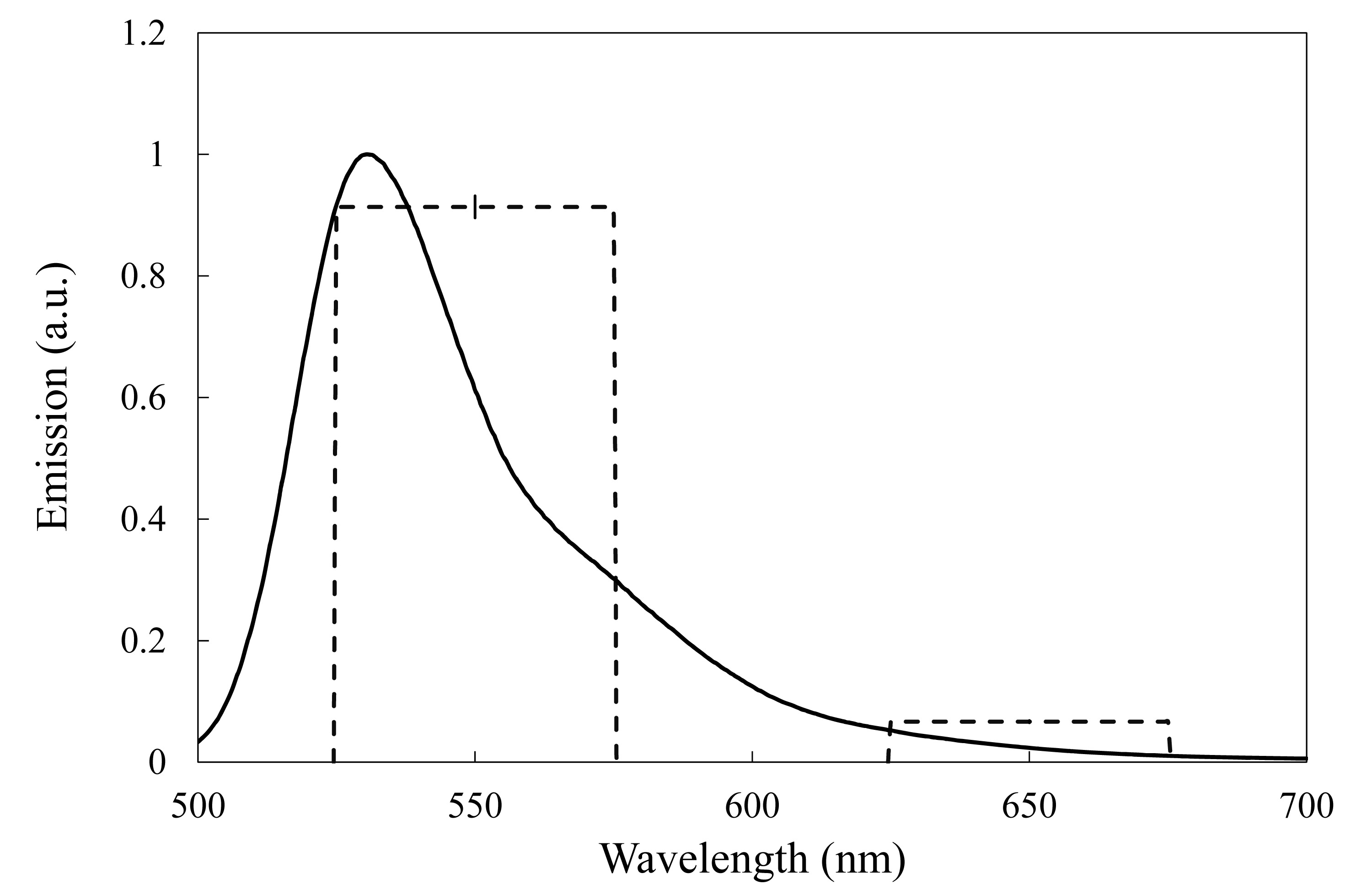

Supplement: S1 Fig — (Dashed line) Delayed Luminescence emission spectrum of an aqueous solution of Rhodamine 123 (0.1 mg/mL). Experimental data are normalized taking into account the spectral dependence of filters’ transmittance and PMT quantum efficiency. Average of four independent experiments ± SE. The total emission in the time interval of one decay (0.1 s) was evaluated as 985 ± 14 counts. For comparison (solid line) the Fluorescence emission spectrum of Rhodamine 123 dissolved in ethanol, when excited at 480nm, it is reported [elaborated from: http://omlc.org/], normalizing every point to the maximum intensity value at λem = 530 nm. Taking into account the differences in DL and F signal intensities, the spectra are quite similar. (TIF) [file pone.0191861.s001.tif]

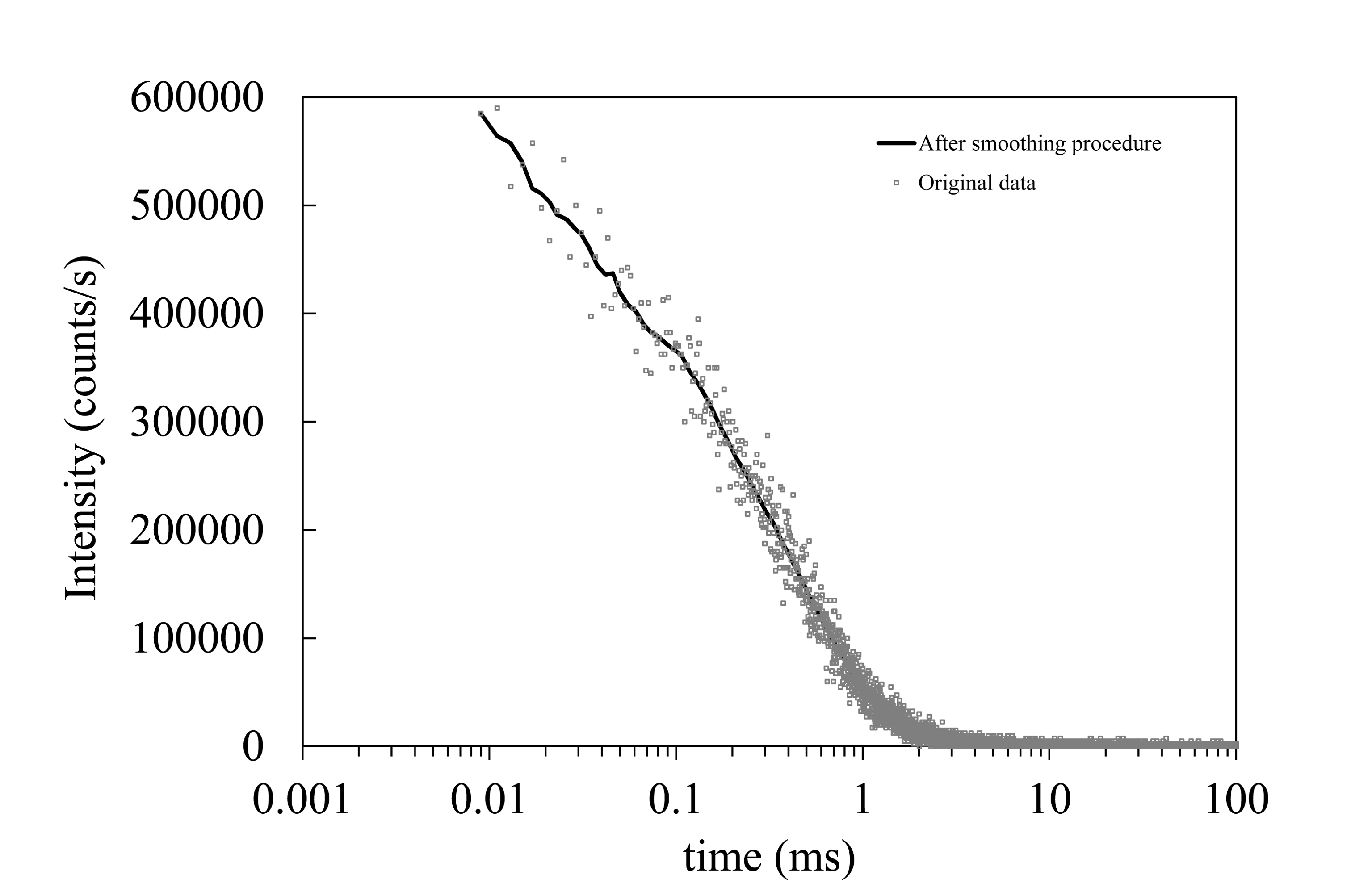

Supplement: S2 Fig — Statistical variations of DL decay from glycerol sample (xg = 1) at 20°C and underlying temporal trend after smoothing procedure. (TIF) [file pone.0191861.s002.tif]

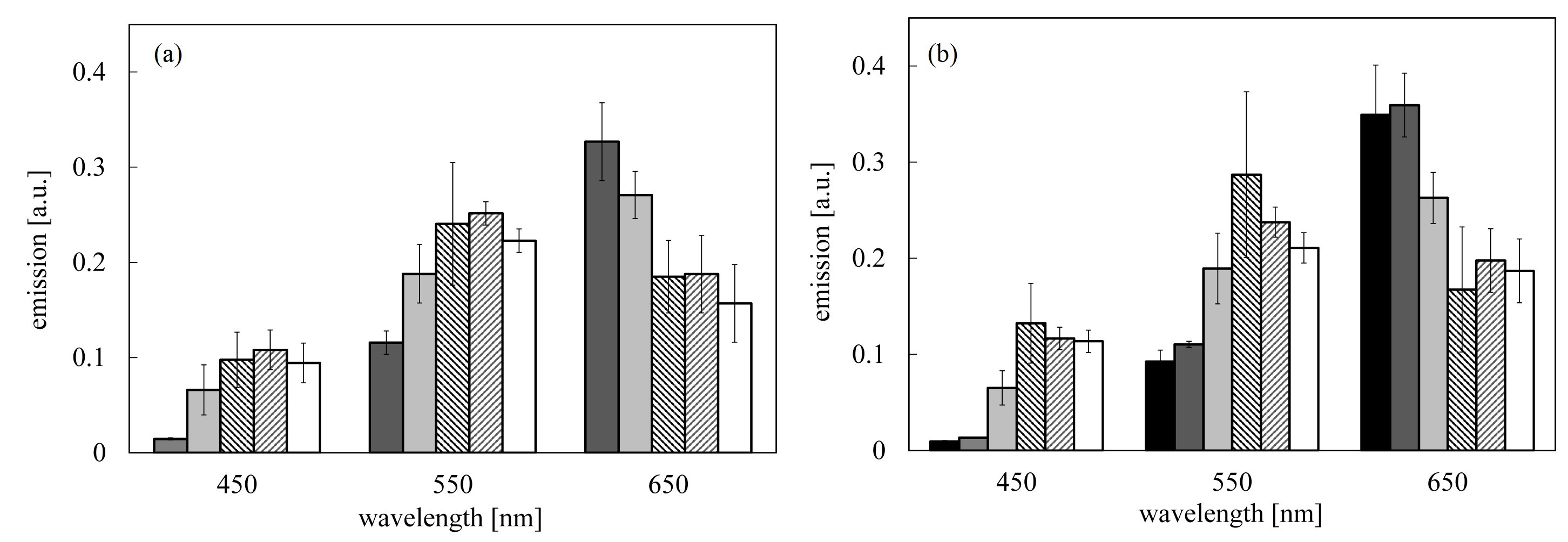

Supplement: S3 Fig — DL emission spectra from samples of different mole fraction xg: (black) xg = 1.00, (dark grey) xg = 0.80, (grey) xg = 0.26, (backward slash) xg = 0.09, (slash) xg = 0.03, (white) xg = 0.00 (water). (a) T = -3°C, (b) T = +8°C. Experimental data are normalized taking into account the spectral dependence of filters’ transmittance and PMT quantum efficiency. Average values and standard deviations are reported. (TIF) [file pone.0191861.s003.tif]
